# Identifying and combating the impacts of COVID-19 on malaria

**DOI:** 10.1186/s12916-020-01710-x

**Published:** 2020-07-30

**Authors:** Stephen J. Rogerson, James G. Beeson, Moses Laman, Jeanne Rini Poespoprodjo, Timothy William, Julie A. Simpson, Ric N. Price, the ACREME Investigators, Nicholas Anstey, Freya Fowkes, James McCarthy, James McCaw, Ivo Mueller, Peter Gething

**Affiliations:** 1 Department of Medicine at the Peter Doherty Institute for Infection and Immunity, University of Melbourne, Melbourne, Australia; 2 Burnet Institute, Melbourne, Australia; 3 Central Clinical School, Monash University, Melbourne, Australia; 4 Department of Microbiology, Monash University, Clayton, Australia; 5 Papua New Guinea Institute of Medical Research, Madang, Madang Province Papua New Guinea; 6 Timika Malaria Research Program, Papuan Health and Community Development Foundation, Timika, Papua Indonesia; 7 Mimika District Health Authority, Timika, Papua Indonesia; 8 Pediatric Research Office, Department of Child Health, Faculty of Medicine, Public Health and Nursing, Universitas Gadjah Mada, Yogyakarta, Indonesia; 9 Rumah Sakit Umum Daerah Kabupaten Mimika, Timika, Papua Indonesia; 10 Infectious Diseases Society Sabah-Menzies School of Health Research Clinical Research Unit, Kota Kinabalu, Sabah Malaysia; 11 Gleneagles Hospital, Kota Kinabalu, Sabah Malaysia; 12 Centre for Epidemiology and Biostatistics, Melbourne School of Population and Global Health, University of Melbourne, Melbourne, Australia; 13 Global and Tropical Health Division, Menzies School of Health Research, Charles Darwin University, Darwin, Australia; 14 Mahidol-Oxford Tropical Medicine Research Unit (MORU), Faculty of Tropical Medicine, Mahidol University, Bangkok, Thailand; 15 Centre for Tropical Medicine and Global Health, Nuffield Department of Clinical Medicine, University of Oxford, Oxford, UK

**Keywords:** Malaria, COVID-19, *Plasmodium*, Elimination, Drug resistance

## Abstract

**Background:**

The COVID-19 pandemic has resulted in millions of infections, hundreds of thousands of deaths and major societal disruption due to lockdowns and other restrictions introduced to limit disease spread. Relatively little attention has been paid to understanding how the pandemic has affected treatment, prevention and control of malaria, which is a major cause of death and disease and predominantly affects people in less well-resourced settings.

**Main body:**

Recent successes in malaria control and elimination have reduced the global malaria burden, but these gains are fragile and progress has stalled in the past 5 years. Withdrawing successful interventions often results in rapid malaria resurgence, primarily threatening vulnerable young children and pregnant women. Malaria programmes are being affected in many ways by COVID-19. For prevention of malaria, insecticide-treated nets need regular renewal, but distribution campaigns have been delayed or cancelled. For detection and treatment of malaria, individuals may stop attending health facilities, out of fear of exposure to COVID-19, or because they cannot afford transport, and health care workers require additional resources to protect themselves from COVID-19. Supplies of diagnostics and drugs are being interrupted, which is compounded by production of substandard and falsified medicines and diagnostics. These disruptions are predicted to double the number of young African children dying of malaria in the coming year and may impact efforts to control the spread of drug resistance. Using examples from successful malaria control and elimination campaigns, we propose strategies to re-establish malaria control activities and maintain elimination efforts in the context of the COVID-19 pandemic, which is likely to be a long-term challenge. All sectors of society, including governments, donors, private sector and civil society organisations, have crucial roles to play to prevent malaria resurgence. Sparse resources must be allocated efficiently to ensure integrated health care systems that can sustain control activities against COVID-19 as well as malaria and other priority infectious diseases.

**Conclusion:**

As we deal with the COVID-19 pandemic, it is crucial that other major killers such as malaria are not ignored. History tells us that if we do, the consequences will be dire, particularly in vulnerable populations.

## Background

The impact of the COVID-19 pandemic on the control of infectious diseases is substantial, undermining established programmes addressing HIV, tuberculosis and malaria and childhood vaccination. This opinion piece focuses on the threat COVID-19 poses to the control of malaria and the steps that can be taken to mitigate these impacts.

Over the past 20 years, major gains have been made in reducing the global burden of malaria, with 11 countries achieving malaria elimination. These gains are largely attributable to expanding the distribution of insecticide-treated bed nets (ITNs), indoor spraying of residual insecticides (IRS) and other vector control strategies; access to early diagnosis (e.g. rapid diagnostic tests (RDTs)); and more effective antimalarial treatments [1], together with targeted interventions such as intermittent preventive treatment in pregnancy (IPTp) and seasonal malaria chemoprevention (SMC). This multipronged approach has been enabled by a greater political, financial and global commitment to malaria elimination, encouraged by ambitious targets, such as reducing malaria globally by > 90% by 2030 (compared to 2015), eliminating malaria from the Asia Pacific by 2030 and Africa being largely malaria-free by 2050.

However, in recent years, progress in reducing the global burden of malaria has stalled. In 2018, there were an estimated 228 million cases, compared with 214 million in 2015, and over 400,000 deaths [2]. Challenges in achieving malaria elimination include the emergence and spread of drug-resistant parasites and insecticide-resistant mosquitos, suboptimal RDTs, lack of universal access to malaria prevention and treatment and the lack of a highly effective vaccine. Malaria funding is below what is required to achieve global goals, and many countries face competing health priorities in the context of severely constrained resources [3]; tuberculosis, human immunodeficiency virus (HIV) infection and other diseases face similar challenges. In this environment, the emergence and spread of COVID-19 presents a huge threat to malaria control that could reverse recent gains in many malaria-endemic countries.

## Learning from prior impacts of disruptions to malaria control

Historically, curtailing malaria control activities has been followed by resurgence in malaria morbidity and mortality [4]. This has occurred when programmes were reduced due to funding constraints or disrupted by war, disaster or conflict [5]. Following the termination of a dichlorodiphenyltrichloroethane (DDT) programme in Indonesia in the 1960s, annual malaria cases rose from < 6000 to 346,000. Between 1946 and 1963, Sri Lanka reduced malaria cases from 2.8 million to just 17, but experienced a massive resurgence to over 500,000 cases per year [5]. While Sri Lanka has now successfully eliminated malaria, other Asian countries that are approaching elimination face similar risks of resurgence if current programmes are substantially disrupted by the COVID-19 pandemic.

Economic collapse and health system failure are known to be critical causes of rising morbidity and mortality from infectious diseases, and this commonly spreads beyond national borders. In Venezuela, the recent economic crisis has been accompanied by population movements and major increases in vector-borne diseases, including a fivefold increase in malaria cases [6]; Venezuela now has over half the malaria cases in the Americas [2], and in adjacent regions of Colombia and Brazil, up to 86% of malaria cases are attributed to recent migration [6]. COVID-19 has already caused major disruption to economic activity, which could contribute to malaria resurgence.

During the Ebola fever crisis in West Africa, excess malaria deaths outnumbered total deaths from Ebola [7]. Deaths from HIV infection and tuberculosis also surged [8]. Contributing factors, including deaths of health care workers, overwhelmed health facilities and fear of contracting disease at health services [8, 9], are relevant to COVID-19. The Ebola epidemic disrupted distribution of ITNs and resulted in increased malaria transmission, while poor access to malaria treatment led to dramatic increases in deaths in children [7]. Subsequent modelling studies indicated that additional ITN distribution and introduction of safe monthly mass drug administration (MDA) [10] with dihydroartemisinin-piperaquine each had the potential to reduce malaria deaths during the epidemic by approximately two thirds [7].

Although international donors pledged support for Ebola, significant delays occurred in getting funds on the ground and this contributed to the severity of the epidemic [11]. Likewise, the magnitude and timing of additional support to COVID-19-affected countries is critical. Support for the management of COVID-19 must be combined with support for malaria treatment and prevention programmes; it is likely that this will need to be sustained for years until COVID-19 is brought under control. Key interventions and innovative approaches, such as targeted MDA programmes and enhanced distribution of ITNs, will be critical in preventing dramatic increases in malaria deaths [12], but their implementation and prioritisation will bring logistic and financial challenges given COVID-19 disruptions and the competing needs of other health issues and services.

## Anticipated impacts of COVID-19 on malaria control and burden

While COVID-19 is less often severe in children and pregnant women [13, 14], these groups would bear a disproportionate burden of excess malaria mortality arising from COVID-19-related disruption of health systems and malaria control programmes, particularly in sub-Saharan Africa. Recently, the Malaria Atlas Project modelled these potential impacts in Africa for the World Health Organization’s (WHO) Global Malaria Program [15]. A range of scenarios were considered, such as ceasing ITN distribution campaigns planned for 2020, reductions of routine ITN distribution and reduced access to effective antimalarial drugs. In the worst-case scenario, a 75% decrease in ITN distribution coupled with a 75% decrease in access to artemisinin combination therapies (ACTs) was predicted to result in a 22% increase in malaria cases, and doubling of malaria deaths within a year to 769,000 [15], 70% of them in children under 5.

These models do not include additional increases in malaria that could result from disruptions in distribution of SMC (which currently protects 19 million children in 12 countries) and IPTp (which protects pregnant women and their babies in 36 African countries from malaria in pregnancy and low birth weight) [2]. Neither do they include the potential impacts of decreasing IRS and other vector control strategies, or the consequences of reassigning malaria personnel to COVID-19-related activities.

The future severity of the impact of COVID-19 in Africa is unknown. At the time of writing, Africa has 4% of the world’s COVID-19 cases, and 2% of its deaths [16], with around 400,000 infections and 10,000 deaths reported [17]. These numbers are likely to be under-estimates due to limited testing being undertaken in some regions. These rising numbers are occurring despite many countries taking early, decisive steps to lock-down borders and implement social distancing in crowded environments and other preventive measures. If these measures, or the disease itself, substantially impede normal functions of the health system, this could result in delayed treatment for young children, in whom severe malaria develops rapidly, even with prompt treatment 10–20% of children with cerebral malaria die. If treatment is not available, staggering numbers of young children may lose their lives from malaria.

COVID-19-related lockdowns threaten the livelihoods of the many Africans who work in the informal sector, affecting their ability to pay for transport and for health care services when these are not free [16, 18]. Preventive and therapeutic maternal child health services such as antenatal clinics and childhood vaccination programmes (with > 80 million infants at risk) are at risk, and economic disruptions may exacerbate child undernutrition [19]. Continued provision of chronic medications for tuberculosis and HIV (there are > 20 million people living with HIV and AIDS in Africa) and access to malaria treatment and prevention services (such as ITN distribution, IPTp and SMC) collectively threaten major increases in infectious disease morbidity and mortality.

## Malaria in Asia: containing drug resistance is critical

While the greatest burden of malaria is in sub-Saharan Africa, two billion people in the Asia Pacific region remain at risk of malaria [2]. This region has been the global hotspot for emergence of *Plasmodium falciparum* resistant to drugs, including chloroquine, antifolates and mefloquine. The spread of chloroquine and antifolate resistance to Africa reversed gains of intense malaria control activities undertaken between 1950 and 1960, leading to increasing mortality and morbidity [20].

In the 1990s, the prospect of untreatable malaria was averted by the introduction of ACTs, which have now been adopted as first-line treatment by almost all malaria-endemic countries [21]. Over the last decade, artemisinin-resistant *P. falciparum*, originating from western Cambodia, has spread across the Greater Mekong Subregion (GMS). The efficacy of a key partner drug, piperaquine, has also declined dramatically [22, 23], causing the efficacy of dihydroartemisinin-piperaquine to fall below 50% in Cambodia, Thailand and Vietnam [24], once again raising concerns of resurgent and untreatable malaria. While new antimalarial agents are urgently required, these are unlikely to be available for several years [25]. WHO has a detailed strategy to contain artemisinin resistance, and substantial resources have been made available to eliminate drug-resistant malaria before it spreads beyond the GMS. From 2000 to 2015, across WHO’s South East Asian Region, there was a 46% reduction in malaria morbidity and a 60% reduction in mortality. These gains remain fragile and depend on robust health systems achieving early diagnosis and treatment of symptomatic patients and clearing parasite reservoirs from asymptomatic populations. If efforts to eliminate drug-resistant *P. falciparum* from the GMS falter, resurgence is highly likely to be followed by the spread of resistance into South Asia, increasing the risks that resistant malaria will spread to Africa.

## Particular challenges of managing malaria during COVID-19

In many places, community-based malaria workers work in close proximity to febrile patients and are at high risk of COVID-19. Despite the risks, adequate personal protective equipment is often lacking, and workers suffer the stigma of being potential sources of viral infection. Funds and personnel are being reassigned from malaria and other programmes to enable COVID-19 response efforts. Malaria elimination campaigns must reach marginalised groups living in remote and border areas [26], but these programmes are at particular risk of being scaled back for logistic or economic reasons associated with COVID-19, putting communities at risk. Together, these complex issues are compromising the provision of health care and surveillance for malaria and threatening elimination efforts.

## Reducing the immediate impacts of COVID-19

To reduce the impact of COVID-19 disruptions, it is essential that the supply of diagnostics and treatments for malaria are maintained and that there is strong support of ITN distribution, IRS and other preventive interventions. Maintaining drug quality is also critical, with potential proliferation of substandard and falsified medicines and diagnostics when supply chains for established suppliers are disrupted [27]. Furthermore, companies may switch from producing drugs or diagnostics for malaria to COVID-19, driven by higher profit margins [28] with clear negative consequences for malaria treatment and control.

Community education and engagement will be important to reinforce messages regarding malaria prevention, diagnosis and treatment. Provision of health services that can provide prompt diagnosis and treatment of malaria is critical, together with professional support of health workers to ensure safe working environments and properly resourced facilities. Ideally, these activities would be integrated within the COVID-19 response [29]. Where adequate personal protective equipment or RDTs are not available, presumptive malaria treatment may be required (based on symptoms of fever without another obvious cause) [12]. This brings risks of confounding malaria and COVID-19 and of missing other key diseases like childhood pneumonia. If health systems are not able to maintain malaria control interventions while managing the response to the COVID-19 emergency, they are highly likely to be further impacted by additional malaria cases.

## Lessons learnt from malaria elimination campaigns

A series of case studies prepared for the WHO in 2012 by the University of California San Francisco [30] documented effective strategies used to re-establish malaria control and support progress towards elimination in 10 countries. Political will is a critical component, with programmes supported by national governments, although implementation may be also be coordinated at local levels. While some countries are able to self-fund flexible programmes, many rely on donor support, particularly from the Global Fund for AIDS, Tuberculosis and Malaria, to assist with procuring diagnostics, drugs and ITNs. Distribution channels must be strengthened, and capacity building is required to ensure that a skilled and adaptable workforce can deliver interventions. Integrated approaches to control vector-borne diseases are essential, with cadres of health care workers benefiting from donor-supported malaria-specific training and other professional development [30]. With regard to service delivery, strengthening the treatment and prevention of malaria must go hand-in-hand with overall health systems strengthening, as part of integrated models of primary health care.

In areas of heterogeneous transmission, robust surveillance and targeted control activities can be highly effective. In countries approaching elimination, such responses will need to include localised vector control responses with focal IRS campaigns and potentially other measures such as environmental management [30]. Intensified passive case detection and prompt disease notification can in turn facilitate targeted screening and active case detection programmes. A combination of reactive focal mass drug administration with artemether lumefantrine and reactive IRS with primiphos-methyl was recently trialled in a low transmission area of Namibia; each intervention halved clinical malaria cases, and the combination reduced malaria by 75% [31].

Management of malaria in border areas requires special initiatives. In some island nations, all arrivals from endemic countries are recorded and monitored, while countries in the end stages of elimination, such as Bhutan, Turkey and Turkmenistan, have developed systems to track and identify infections in mobile populations including cross border traders, migrant workers and refugees. Transnational cooperation around borders is key to tackling malaria in undocumented migrants, seasonal workers and marginalised populations in remote border areas. Economic disruption following the COVID-19 pandemic is likely to lead to increases in malaria cases or changing patterns of population movements in these groups.

In high transmission areas, continuing malaria surveillance combined with real-time reporting will allow the prompt detection of hotspots of transmission. ITN distributions and IRS campaigns combined with MDA hold great potential for bringing malaria under control. Technical advances such as Geographical Information System (GIS)-based case mapping and electronic reporting of cases can provide real-time data on numbers and locations of cases and tailored responses to high burden areas. In the context of COVID-19, surveillance may be difficult due to multiple factors, including restrictions on movements, concerns about field worker exposure, requirements for additional PPE or resource constraints. Therefore, integrated, population-wide approaches that include ITN distribution and MDA and/or IRS for malaria with treatment of neglected tropical diseases, and community health education, may be more practicable to maximise population protection.

Many successful malaria elimination campaigns have had a strong focus on community awareness and community participation. The latter may extend to active involvement in environmental management and breeding site interventions (as in Réunion), and there is great potential to integrate community education and health promotion on malaria with COVID-19 activities. Recent years have seen the emergence of civil society organisations such as the Civil Society for Malaria Elimination [32] and campaigns such as #ZeroMalariaStartsWithMe, which are empowering community and civil society to work for effective, sustainable and inclusive malaria control solutions. Close engagement with local community is integral to the implementation of high-impact programmes.

Private sector involvement is important on several levels. Private clinics, pharmacies and local drug sellers have crucial roles in ensuring accurate diagnosis and prompt effective treatment of malaria. In many settings, these services also have important roles in reporting malaria infection [30]. Large enterprises in extractive and agricultural industries may house great numbers of workers and families, sometimes on a seasonal basis. These can be sources of local disease outbreaks or can help to strengthen the surrounding health care system and work jointly on malaria control activities. Irrigation schemes require ongoing maintenance and oversight to prevent the development of vector breeding sites. Partnerships between successful local companies and local health authorities can help to support specific malaria control activities of mutual interest, to “give back” to their home community.

## Rebuilding malaria control after the first wave

Following the first COVID-19 epidemic peak in malaria-affected countries, reinvigoration of malaria control will be needed in many regions (Table 1), and steps will need to be taken to prepare for further waves of infection. Strengthening surveillance for malaria is crucial together with campaigns to ensure sustainable provision of antimalarials and ITNs, while strategies are explored to allow community-based malaria programmes to continue. These efforts will inform health system responses. Lessons can be learnt from childhood immunisation programmes, which have achieved significant successes in increasing immunisation coverage following disruptions through programme intensifications, outreach activities, community engagement and political support and leadership. Transnational and regional initiatives, including operational research, will help to identify strategies for subsequent malaria control activities.

**Table 1:** Priorities for combating malaria

Ensure the safety of health care workers and the populations they serve through adequate provision of personal protective equipment, hand hygiene and ability to practice social and physical distancing.	
Provide resources to enable national malaria control programmes to continue to carry out established programmes.	
Maintain campaigns and systems to procure and distribute ITNs, ensuring continuing coverage of high-risk populations.	
Secure ongoing production and supply chains of quality-approved malaria diagnostics, treatments and preventives.	
Ensure the timely delivery of these essential supplies to all health facilities.	
Consider safely implementing campaigns of mass drug administration, especially during periods of peak malaria risk.	
Support malaria-endemic countries both in fighting COVID-19 disease and in controlling malaria through an integrated health care programme and community engagement.	
Resume, and maintain funding for, non-COVID-19 research, from discovery through to clinical, epidemiological and health system studies [33].	
Maintain existing global funding for malaria control and elimination.	
Recommit to the elimination of malaria from the Asia Pacific and renew efforts to achieve a malaria-free world.	

## Conclusion

As we struggle with COVID-19, we call on the leaders of countries across the world to recommit to malaria elimination as an achievable and enduring public good. The broader goal of malaria eradication remains highly attractive. An estimated US$90–$120 billion investment would unlock over US$2 trillion in economic benefits [34]. If we reduce our focus on malaria, it will resurge, bringing a terrible death toll and even greater economic hardship. If efforts to eliminate drug-resistant *P. falciparum* malaria from the GMS falter, resistant malaria will likely spread to Africa, an outcome that could lead to a dramatic increase in childhood deaths [24].

The world’s most affluent countries are already grappling with the challenges of delivering health care and responding to the secondary social and economic costs of the COVID-19 pandemic. In malaria-endemic countries, it is vital that measures are taken both to protect health workers and maintain malaria control activities. The global community cannot afford to cut aid to established programmes that have contributed significantly to the major progress made against malaria and other diseases; we must continue to look outwards, rather than turning inwards at the expense of the world’s most vulnerable. Successful malaria programmes that are depleted by the COVID-19 epidemic must be rebuilt as quickly as possible to prevent a novel pathogen from giving a new lease of life to an old one.

## Data Availability

Not applicable

## Abbreviations

ACT: Artemisinin combination
AIDS: Acquired immune deficiency syndrome
COVID-19: Coronavirus disease
GIS: Geographical Information Systems
GMS: Greater Mekong Subregion
HIV: Human immunodeficiency virus
IPTp: Intermittent preventive treatment in pregnancy
IRS: Indoor spraying of residual insecticides
ITN: Insecticide-treated net
MDA: Mass drug administration
RDT: Rapid diagnostic test
SMC: Seasonal malaria chemoprevention
WHO: World Health Organization
